# Nano-Stenciled RGD-Gold Patterns That Inhibit Focal Contact Maturation Induce Lamellipodia Formation in Fibroblasts

**DOI:** 10.1371/journal.pone.0025459

**Published:** 2011-09-27

**Authors:** Roman Lutz, Kristopher Pataky, Neha Gadhari, Mattia Marelli, Juergen Brugger, Matthias Chiquet

**Affiliations:** 1 Friedrich Miescher Institute for Biomedical Research, Basel, Switzerland; 2 Microsystems Laboratory, Ecole Polytechnique Fédérale de Lausanne, Lausanne, Switzerland; 3 Department of Orthodontics and Dentofacial Orthopedics, University of Bern, Bern, Switzerland; Leiden University, The Netherlands

## Abstract

Cultured fibroblasts adhere to extracellular substrates by means of cell-matrix adhesions that are assembled in a hierarchical way, thereby gaining in protein complexity and size. Here we asked how restricting the size of cell-matrix adhesions affects cell morphology and behavior. Using a nanostencil technique, culture substrates were patterned with gold squares of a width and spacing between 250 nm and 2 µm. The gold was functionalized with RGD peptide as ligand for cellular integrins, and mouse embryo fibroblasts were plated. Limiting the length of cell-matrix adhesions to 500 nm or less disturbed the maturation of vinculin-positive focal complexes into focal contacts and fibrillar adhesions, as indicated by poor recruitment of α5-integrin. We found that on sub-micrometer patterns, fibroblasts spread extensively, but did not polarize. Instead, they formed excessive numbers of lamellipodia and a fine actin meshwork without stress fibers. Moreover, these cells showed aberrant fibronectin fibrillogenesis, and their speed of directed migration was reduced significantly compared to fibroblasts on 2 µm square patterns. Interference with RhoA/ROCK signaling eliminated the pattern-dependent differences in cell morphology. Our results indicate that manipulating the maturation of cell-matrix adhesions by nanopatterned surfaces allows to influence morphology, actin dynamics, migration and ECM assembly of adhering fibroblasts.

## Introduction

Cell adhesion to extracellular matrix (ECM) is mediated mainly via integrins, heterodimeric cell surface receptors that play key roles in transmembrane signaling processes and thereby regulate cell behavior and fate [1]. One important group of integrins interacts with Arg-Gly-Asp (RGD) peptide motifs that are presented by many ECM ligands; depending on their subunit composition, however, these receptors bind to individual RGD-containing ECM proteins with different affinities. For example, the vitronectin receptor integrin-αvβ3 recognizes RGD in various contexts (also in short peptides), whereas integrin-α5β1 very specifically interacts with fibronectin. Intracellularly, integrins are linked to the actin cytoskeleton by means of specialized adaptor proteins such as talin and vinculin, which form a dense and heterogeneous protein network [2]. Interaction partners also involve protein kinases like Src and FAK that build a platform for early steps of signaling. Since cell-matrix adhesions are the sites where forces are transmitted from the ECM to the cytoskeleton and back, they are critical for transducing mechanical stimuli, such as substrate rigidity or changes in substrate strain, into chemical signals [1]. Among the signaling cascades that are activated by mechanical stimuli is the RhoA/ROCK pathway [3], [4] that promotes actin stress fiber formation [5], [6].

Attachment and spreading of cells on an ECM substrate occurs in several steps that depend on integrin-mediated sensing of local substrate features, a process which in turn controls the formation and maturation of cell-matrix adhesions. Shortly after first contact of a cell with its substrate, Rac1, a Rho-family GTPase, stimulates the assembly of a fine meshwork of actin filaments at cell borders and the protrusion of lamellipodia [7], [8]. A lamellipodium represents the “leading edge” of a moving or spreading cell and is the birthplace of cell-matrix adhesions called focal complexes. These small, dot-like adhesions are less than 1 µm^2^ in area and characterized by the colocalization of αvβ3-integrin, paxillin, talin, vinculin, FAK and phosphotyrosine [9], [10], [11]. Focal complexes are highly ephemeral and often disassemble rapidly. Alternatively, if at this site the ECM substrate is adhesive (i.e. of high integrin ligand density) and mechanically stable, focal complexes can mature into focal contacts by growing in size and recruiting additional proteins like zyxin, tensin, and α5β1-integrin. This maturation depends on actin and myosin-II induced cellular traction and can be arrested by inhibitors of the RhoA/ROCK pathway [10], [12]. Mature focal contacts are usually found at the tip of actin stress fibers; they are 1–2 µm wide, several µm long, and can persist for many minutes at the same location. Further traction by means of the actin cytoskeleton pulls out α5β1-integrin and tensin from focal contacts, initiating the formation of fibrillar adhesions that are tens of µm long [13]. During this process, secreted pericellular fibronectin is bound to α5β1-integrin and stretched, and exposes self-assembly sites that lead to the formation of fibronectin fibrils [14]. Thus, on most substrates the different types of cell-matrix adhesions are assembled in a hierarchical way, growing in size during the process.

Much has been learned about the mechanisms of integrin-dependent cell-ECM interactions by the use of micro- and nanopatterened adhesive substrates that were engineered in recent years [15], [16], [17]. The most widely used method is microcontact printing, whereby patterns of adhesive ECM proteins (e.g. fibronectin) are applied onto a passivated (non-adhesive) background surface. Cell-sized ECM islands of various shapes were designed to impose onto cells a defined surface area and form; this allowed to study how cell shape controls cell survival [18], division [19], and differentiaton [20]. On the other hand, ECM stripe or dot patterns in the micro- and nanometer range were engineered that forced cells to establish cell-matrix contacts only at locations defined by the print design. With these techniques, essential physical parameters of ECM substrates could be explored that are required for focal contact formation [16], cell spreading [21], cell polarity and cell motility [22].

In the present study, we used a different technique to produce substrate patterns in the sub-micrometer range. By a nanostencil procedure [23], gold squares were applied to glass surfaces, functionalized with RGD-thiol peptide, and the intervening spaces passivated with poly(L-lysine)-graft-poly(ethylene glycol) (PLL-g-PEG). Because of the almost covalent nature of the Au-S bond, organic thiol compounds assemble into monolayers on gold surfaces [24], and it is thus generally assumed that the amount of coupled RGD-thiol is proportional to the gold surface area [16]. RGD rather than fibronectin was used here as a ligand, in order to render the initial cell adhesion dependent on integrin-αvβ3. Cells that attach to a pure fibronectin substrate immediately engage integrin-α5β1; this leads to activation of RhoA [25] and the distinction between different types of cell-matrix adhesions becomes blurred [6]. For the same reason, medium depleted of exogenous fibronectin was used to plate cells on the nanopatterns. The smallest RGD-gold pattern consisted of squares 250×250 nm (0.06 µm^2^) in size, spaced 250 nm apart. For the larger pattern, the respective dimensions were 500, 1000, and 2000 nm. On purpose, for all patterns used in these experiments the coverage of substrate area with integrin ligand (i.e. RGD-coupled gold) was set to 25%, which is far in excess to that reported to be required for full cell spreading [21]. We used this new method with the aim to restrict the size of cell matrix adhesions, and hence to inhibit the maturation of small, newly formed focal complexes into larger, α5β1-integrin-positive focal contacts. Similar nanopatterns to control focal adhesion assembly have been produced before by electron beam lithography [26]; however, downstream effects on cell behavior were not explored in this study. Here, we asked specifically how manipulating the size and spacing of cell-matrix adhesions affected cell shape and motility. We found that cells were able to attach and spread on sub-micrometer RDG-gold patterns via small focal complexes, but that the formation of focal and fibrillar adhesions was inhibited. The nanostructure of cell-matrix adhesions in turn affected lamellipodia formation, the actin cytoskeleton, fibronectin fibrillogenesis, and directed cell migration. Our results suggest that distinct signals arise from cell-matrix adhesions during different stages of their maturation.

## Results

### Design of the RGD-gold patterns

**Figure 1:**
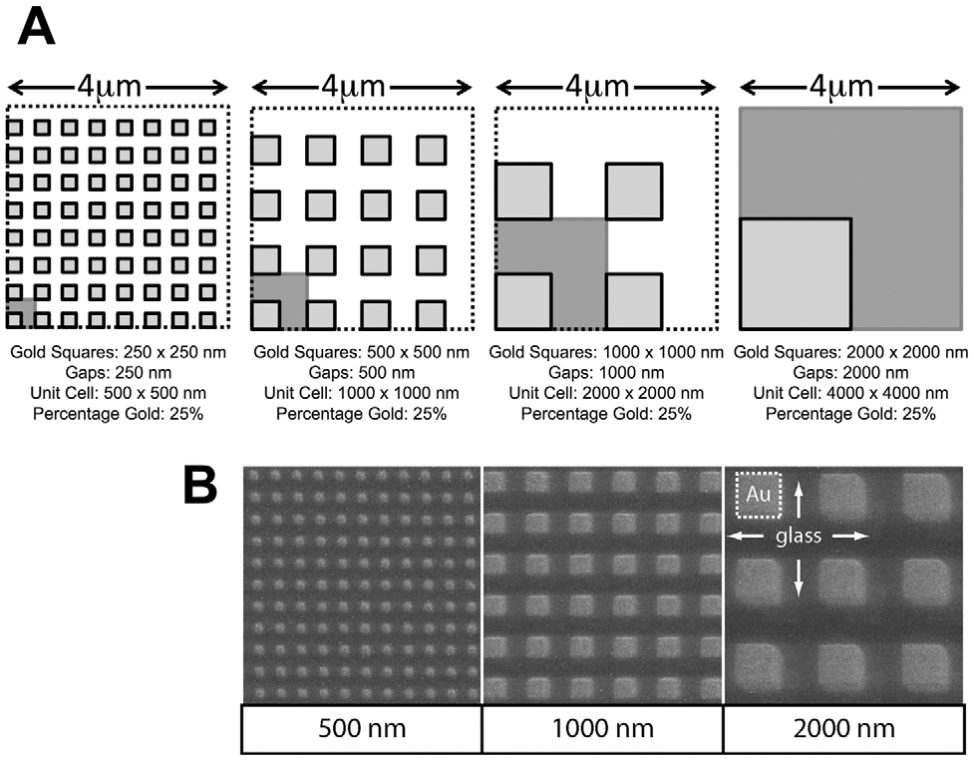
RGD-gold patterns produced by the nanostencil technique. (A) Design of the patterns. Gold squares of the indicated sizes were applied to glass surfaces (see Materials and Methods). Unit cells of the four individual patterns are shown in blue. Note that by keeping the width of the squares the same as their lateral spacing, the patterns were designed such that the surface of RGD-coupled gold was fixed to 25% of the total area. After manufacturing the patterns, RGD peptide was coupled to the gold squares to provide restricted and integrin-specific cell attachment, and intervening spaces were passivated with PLL-g-PEG. (B) SEM micrographs of patterns of gold squares (500×500 nm^2^, 1000×1000 nm^2^ and 2000×2000 nm^2^) on glass coverslips. The 250×250 nm^2^ squares could not be imaged due to electron charging effects.

In order to restrict the growth of cell-matrix adhesions during cell adhesion to a defined size, gold square patterns in the micrometer and sub-micrometer range were applied to glass surfaces with a nanostencil technique [23]. Before seeding fibroblasts, Cys-containing RGD peptide was bound to the gold squares to provide restricted and integrin-specific cell attachment, and the area surrounding the gold squares was passivated with poly-L-lysine covalently grafted to polyehthylene glycol (PLL-g-PEG; [27]). Four different patterns with gold squares ranging from 250×250 nm^2^, 500×500 nm^2^, 1×1 µm^2^ to 2×2 µm^2^ were prepared (Fig. 1). To ensure that cells on all patterns had the same areal density of adhesive ligand to attach, the patterns were designed in a way that the surface area of RGD-coupled gold was fixed to 25% of the total substrate area. This was achieved by spacing the gold squares at a distance corresponding to their side length (Fig. 1A). Control experiments showed that gold surfaces were functionally saturated with RGD peptide under the coating conditions used (see Materials and Methods; Figure S1). Thus, the only difference between the four patterns used is the distribution, not the amount or density, of adhesive RGD-gold ligand. Scanning electron microscope images of the patterns are shown in Fig. 1B. For simplicity, the four different substrates are called 250 nm, 500 nm, 1000 nm and 2000 nm patterns in the following paragraphs. For positive and negative controls, cells were plated on plain (i.e. unpatterned) RGD-gold or on plain PLL-g-PEG passivated glass surfaces, respectively.

### Restriction of cell-matrix adhesions to RGD-coupled gold areas

**Figure 2:**
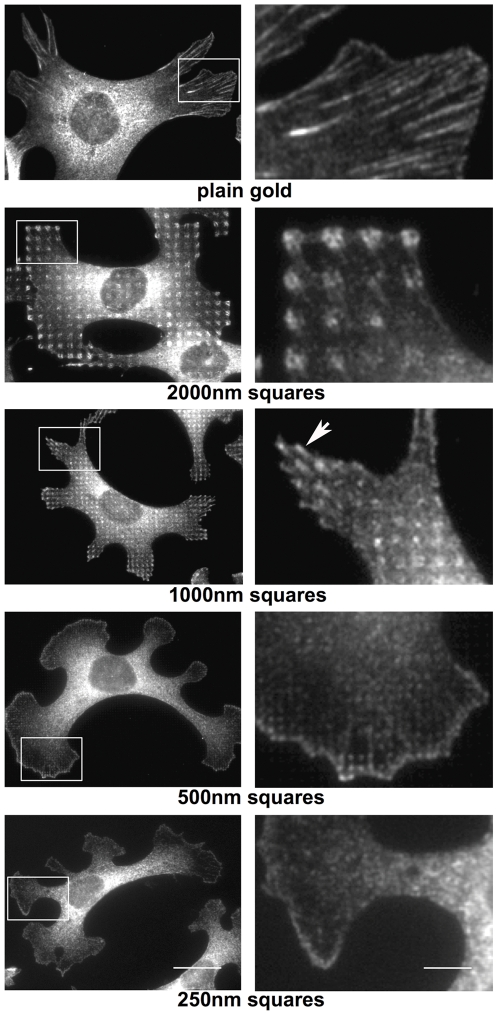
Fibroblasts form matrix adhesions only on RGD-coupled gold areas. Cells were grown on RGD-coupled gold patterns for 4 hours with 3% fibronectin-free serum and subsequently fixed and stained with an anti-vinculin antibody. The images in the right row are enlargements of the boxed areas at left. Note that vinculin staining in cell-matrix adhesions is restricted to RGD-gold nano- and microsquares, whereas on plain RGD-gold vinculin-positive adhesions are several micrometers long. On the 1000 nm pattern, peripheral adhesions over gold squares are elongated in the centripetal direction (arrow). Scale bar is 20 µm (left row) and 5 µm (right row), respectively.

Mouse embryo fibroblasts (MEF) were plated on the RGD-coupled and PLL-g-PEG-passivated gold patterns in medium containing 3% fibronectin-depleted FCS (to reduce nonspecific adhesion). Cells were allowed to attach and spread for 4 hours, subsequently fixed and stained for vinculin (Fig. 2). Some cytoplasmic staining in the perinuclear area was seen on all substrates. On plain RGD-coupled gold surface, specific staining for vinculin was additionally found in elongated, up to 10 µm long cell-matrix adhesions primarily at the cell border, with radial orientation towards the cell center. Such a distribution is indistinguishable from what is observed for the same cells on tissue culture plastic (not shown; see eg. [6]). On the 2000 nm and 1000 nm patterns, anti-vinculin labeled cell-matrix adhesions were located over the RGD-coupled gold squares. More intense staining was usually detected for adhesion sites in the cell periphery; however, they were also present in more central regions. On the 2000 nm patterns, often two vinculin-positive, elongated foci could be discerned on individual RGD-gold squares. In most cases, they ran either parallel or diagonal to the sides of the squares, and their orientation was roughly centripetal with respect to the cell nucleus. On the 1000 nm patterns, a single cell-matrix adhesion practically filled the area of each RGD-gold square. Interestingly, however, many peripheral adhesions exhibited a vinculin-positive short “tail” that extended over the margin of the respective 1 µm^2^ square, pointing again towards the cell center. The directionality of cell-matrix adhesions on both the 2000 nm and 1000 nm patterns indicated that they were subjected to centripetal cytoskeletal force by actin contractility (see below; Fig. 5), a typical feature of classical focal adhesions [1]. In contrast, on the 500 nm patterns vinculin was assembled in dot-like, non-polarized cell-matrix adhesions that were most prominent at the periphery of large lamellipodia, and again matched the underlying RGD-gold squares (Fig. 2). A similar observation was made for cells on the 250 nm RGD-gold dots although this pattern is at the limit of resolution for light microscopy. Thus on all patterns, vinculin-positive cell-matrix adhesions were clearly positioned over areas coated with RGD-coupled gold.

In the absence of gold or without coupling of RGD, cells did neither attach nor spread on the passivated substrate (not shown).

### Poor recruitment of α5β1-integrin to matrix adhesions on RGD-gold squares smaller than 1 µm^2^

**Figure 3:**
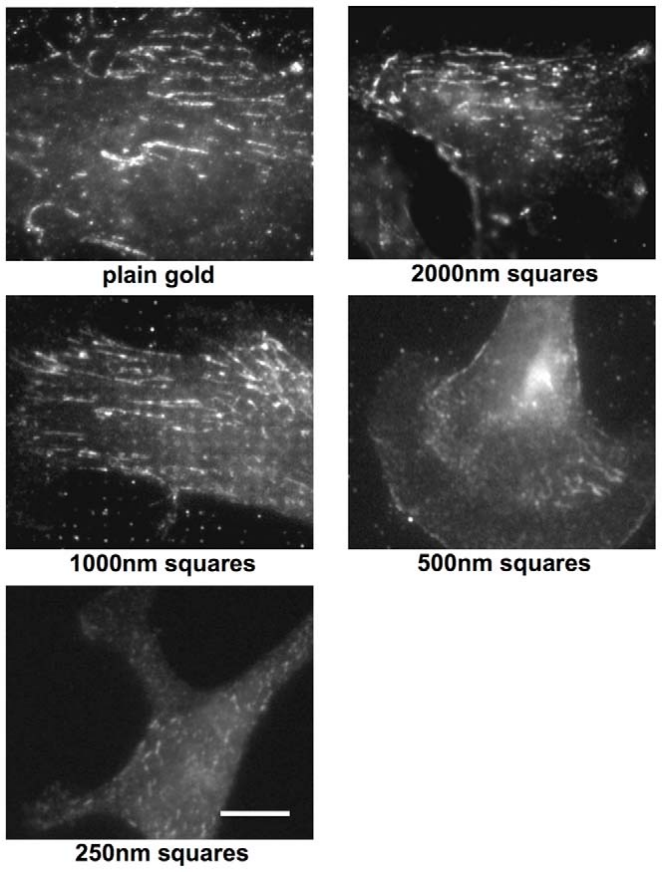
Integrin α5β1 is poorly recruited to matrix adhesions on RGD-gold squares smaller than 1 µm^2^. Cells were grown on RGD-coupled gold patterns for 4 hours with 3% fibronectin free serum and subsequently fixed and stained with an anti-α5-integrin antibody. Notice that α5β1-integrin localizes to fibrillar adhesions on plain gold and on patterns ≥1 µm^2^, whereas on smaller patterns it is only modestly recruited to cell adhesions. Scale bar is 10 µm.

Integrin-α5β1 is largely absent from focal complexes of spreading cells [2], but during their maturation is recruited into early focal adhesions [28] where it binds secreted fibronectin [25]. Shortly later, α5β1-integrin is pulled out of early focal adhesions to form fibrillar adhesions, leaving late focal adhesions behind with a low content of this integrin [13]. To analyze the localization of α5β1-integrin in cell-matrix adhesions, we plated fibroblasts on the various RGD-gold patterns for 4 hours and stained them with anti-α5-integrin antibody (Fig. 3). On plain RGD-gold (as on tissue culture plastic; not shown), cells exhibited many α5-integrin-positive fibrillar adhesions several µm in length, many of them starting at the cell periphery and oriented in the long axis of the cell. A similar distribution was observed on the 2000 nm and 1000 nm patterns: The cells bridged neighboring RGD-gold squares with α5-integrin positive fibrillar adhesions, and hence overcame the restriction given by pattern. However, staining for α5-integrin was often brighter over the RGD-gold dots (Fig. 3), i.e. at sites of vinculin-positive focal adhesions (cf. Fig. 2). In contrast, on RGD-coupled gold patterns with dot size smaller than 1 µm^2^, staining for α5-integrin in cell-matrix adhesions was clearly diminished, especially in peripheral regions of the cells (Fig. 3). A small amount of this integrin subunit was still found more centrally in short (1–3 µm long) fibrillar structures, which however did not appear to correlate with the underlying RGD-gold pattern. These data indicated that the vinculin-positive, α5-integrin-negative structures detected in the cell periphery on 250 nm and 500 nm patterns (Fig. 2) can indeed be classified as focal complexes that failed to be converted into focal and fibrillar adhesions.

### Enhanced lamellipodia formation on RGD-gold squares smaller than 1 µm^2^

**Figure 4:**
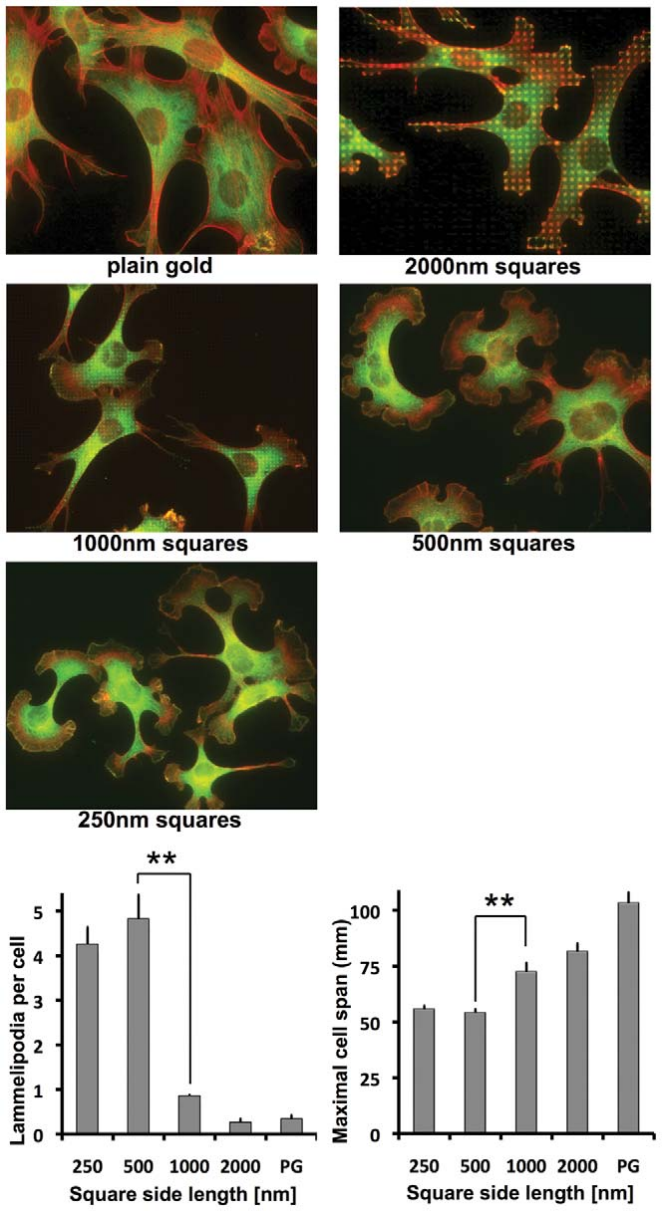
Fibroblasts on RGD-gold squares smaller than 1 µm^2^ show altered morphology. Cells were grown on RGD-coupled gold patterns for 4 hours with 3% fibronectin-free serum and subsequently fixed and stained with Alexa-labeled phalloidin (red) and anti-vinculin antibody (green). Scale bar is 50 µm. Note the high number of lamellipodia on the 250 nm and 500 nm patterns, which is quantified in the graph (bottom left). In addition, the maximal cell span was reduced on the smaller compared to the larger patterns (bottom right). PG = Plain gold. Values indicate the mean ± S.E.M. from three independent experiments including ≥30 cells per pattern size; **p<0.01.

When plating fibroblasts on the various RGD-gold patterns, we found clear differences in cell morphology already by visual inspection with a phase contrast microscope. To analyze cell-shape more quantitatively, we fixed cells after 4 hours, co-stained them for vinculin and actin, and viewed them by fluorescence microscopy. On plain RGD-gold as well as on the 2000 nm and the 1000 nm patterns, cells were well spread 4 hours after plating (Fig. 4). Fibroblasts appeared elongated with usually several (3–7) spiky processes and few lamellipodia (less than one per cell in average). However, on the 500 nm and the 250 nm patterns the cell morphology changed drastically. Cells again were well spread, but assumed a phenotype with an average of 4–5 extended lamellipodia per cell and almost no spike-like processes. Lamellipodia did not localize to a specific end of the cell but rather formed all around the perinuclear cell body. On the 500 nm and the 250 nm patterns, cells were also less elongated with an average long axis of 55 µm. In comparison, the long axis for cells on the larger RGD-gold patterns was measured to be 75–80 µm in average, and it reached about 100 µm on plain gold (Fig. 4).

### Altered actin assembly on RGD-gold squares smaller than 1 µm^2^

**Figure 5:**
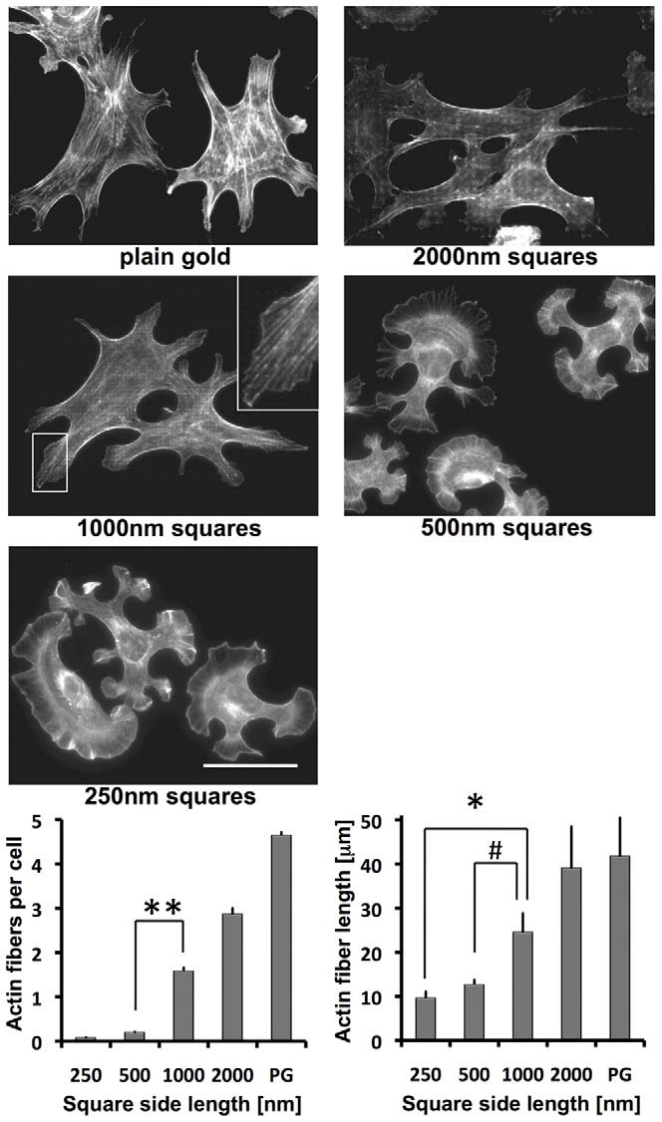
Fibroblasts on RGD- gold squares smaller than 1 µm^2^ do not form actin stress fibers. Cells were grown on RGD-coupled gold patterns for 4 hours with 3% fibronectin free serum and subsequently fixed and stained with Alexa-labeled phalloidin. The insert at the upper right of the cells on the 1000 nm pattern is an enlargement of the boxed area. Scale bar is 50 µm. For quantification of stress fibers, a fluorescence intensity threshold was set to distinguish stress fibers from remaining actin structures. Note the increased number (bottom left) and length (bottom right) of actin stress fibers on patterns ≥1 µm^2^ and on plain gold (PG). Values indicate the mean ± S.E.M. from three independent experiments including ≥30 cells per pattern size; **p<0.01, *p<0.05, #p = 0.056.

We found clear differences in the number of lamellipodia in fibroblasts grown on different RGD-gold patterns, and cells showed a different morphology (Fig. 4). Since actin assembly is largely involved in the formation of cellular processes, we wanted to analyze the actin cytoskeleton more precisely. To this aim, cells grown on the various patterns for 4 hours were stained with Alexa-labeled phalloidin and analyzed by fluorescence microscopy. In fibroblasts on plain RGD-gold substrate, we found thick actin stress fibers throughout the cells, which often bundled in the cell center forming actin foci (Fig. 5). Also on the 2000 nm pattern thick actin stress fibers were identified. Some crossed the cell body but most of them were found along the cell borders. This might be a reason why actin foci were hardly found on these patterns. Fibroblasts on the 1000 nm pattern only exhibited actin stress fibers at the cell border. More central actin fibers were rather fine but still aligned parallel to the major cell axis. They started at the cell margin over gold squares and extended centripetally (see insert in Fig. 5), in accordance with the location and elongated shape of focal adhesions on the 1000 nm pattern (cf. Fig. 2). In contrast, on the 250 nm and 500 nm patterns thick actin stress fibers were basically absent. In some cells, short actin bundles were found at the cell margin perpendicular to the border of lamellipodia. However, most of the actin was polymerized into a fine meshwork in lamellipodia and also in the cell body. At the rear end of protruding lamellipodia, the orientation of the meshwork was parallel to the cell border. To quantify our observations, images from three independent experiments with a total of around 50 cells per pattern were evaluated. The threshold of the 8-bit pictures was set to a grey value of 150, and remaining visible structures with a fibrous shape were counted as actin stress fibers. The number of stress fibers above threshold increased from almost zero on the 250 nm and 500 nm patterns to 2–3 on the 1000 nm and 2000 nm patterns, and up to 5 in average on plain RGD-gold substrate (Fig. 5, lower panel). The mean length of these structures gradually increased from 10 mm on the 250 nm pattern to 40 µm on plain RGD-gold.

### Altered fibronectin fibrillogenesis on RGD-gold squares smaller than 1 µm^2^

**Figure 6:**
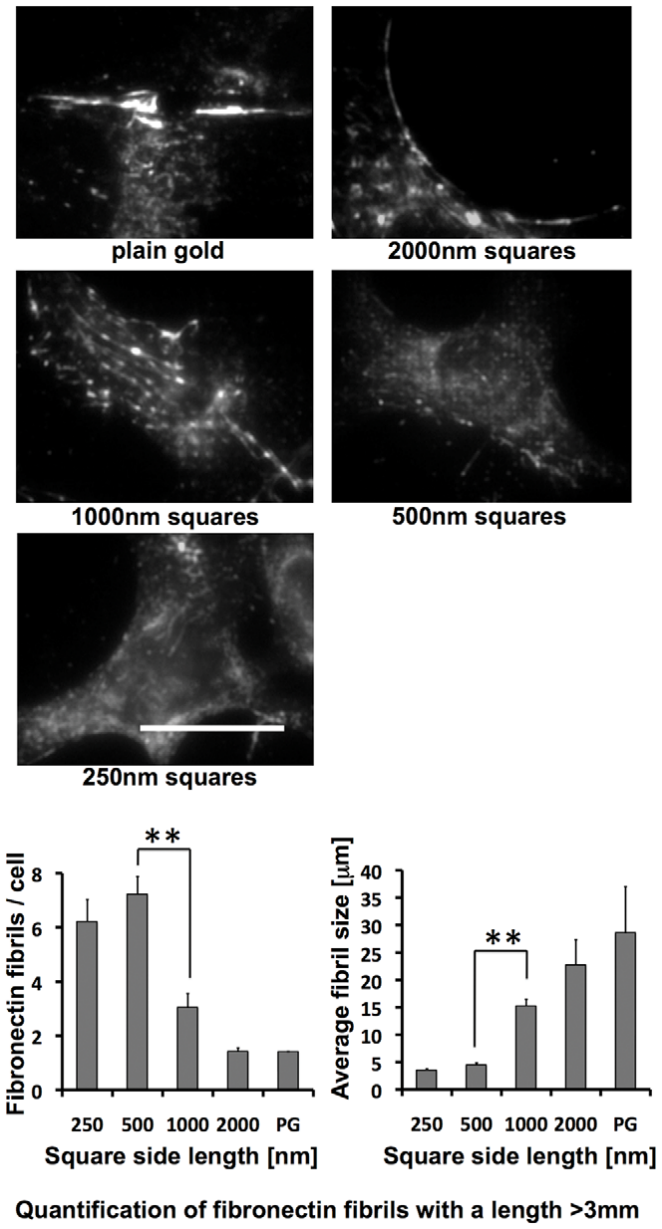
Fibroblasts on RGD-gold squares smaller than 1 µm^2^ form short and thin fibronectin fibrils. Cells were grown on RGD-coupled gold patterns for 4 hours with 3% fibronectin free serum and subsequently fixed and stained with an anti fibronectin antibody. Note that long fibronectin fibrils are detected only on patterns ≥1 µm^2^ and on plain gold (PG). Scale bar is 20 µm. Quantifications of the number (bottom left) and size (bottom right) of fibronectin fibrils with a length >3 µm per cell are shown by the graphs. Values indicate the mean ± S.E.M. from three independent experiments including ≥30 cells per pattern size; **p<0.01.

Fibronectin fibrillogenesis by fibroblasts depends on actomyosin contractility [29]. Since cells on the 250 nm and 500 nm pattern assembled actin into a fine meshwork rather than stress fibers, we asked whether these cells are still able to assemble fibronectin into fibrils. Therefore we cultured cells on RGD-coupled gold patterns in fibronectin-depleted medium as described in Materials and Methods. Cells were fixed 4 hours after plating and subsequently stained with anti-fibronectin antibody (Fig. 6). Within this short time, fibroblasts on plain RGD-gold assembled their secreted fibronectin into a few, relatively thick and long fibrils per cell. The fibrils reached a length of 15–30 µm in average. Long fibronectin fibrils were also observed for cells on the 2000 nm and 1000 nm patterns (Fig. 6). They roughly aligned with the underlying pattern, and as for α5-integrin (c.f. Fig. 3), the staining intensity for fibronectin was increased over the RGD-gold dots. Conversely, on the 500 nm and the 250 nm pattern cells assembled more numerous, but fainter and shorter fibronectin fibrils. To quantify our observations, fibronectin fibrils with a length above 3 µm were scored. On the 250 nm and the 500 nm patterns, cells formed in average 6–7 fibrils with a size longer than 3 µm. A large number of fibrils shorter than 3 µm were observed; however, these were often difficult to distinguish from plaque-like fibronectin aggregates and therefore were not included for quantification. On the 1000 nm and 2000 nm patterns the number of fibronectin fibrils per cell above threshold was lower, whereas their average length was increased several fold (Fig. 6).

### Decreased rate of cell migration on 500 nm versus 2000 nm RGD-gold patterns

**Figure 7:**
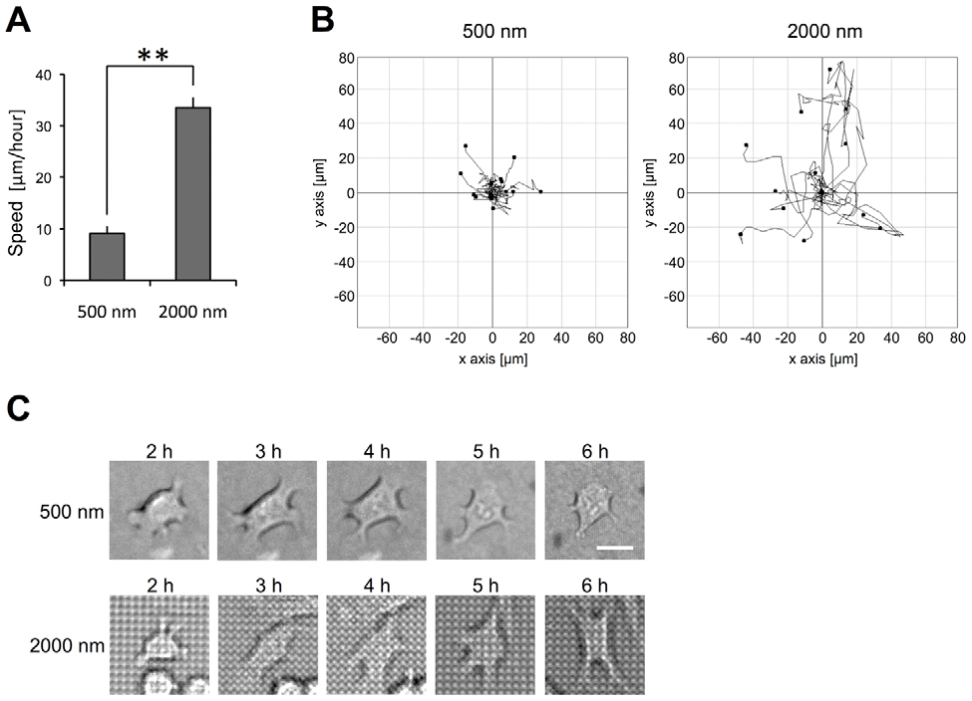
Cell migration speed is decreased on 500 nm versus 2000 nm RGD-coupled gold squares. Fibroblasts were plated on RGD-coupled gold patterns in 3% fibronectin free serum and analyzed by time-lapse microscopy for 6 hours. A) Three hours after plating the cell speed was determined for the following 3 hours. For each pattern (500 nm and 2000 nm) ≥40 cells from three independent experiments were analyzed. Values indicate mean velocity ± S.E.M.; **p<0.01. B) Cell tracking plots on the 500 nm and the 2000 nm patterns for one of the experiments described in A. C) Differential interference contrast pictures of representative cells on each pattern (500 nm and 2000 nm) are shown. The pictures are taken at 2, 3, 4, 5 and 6 hours after plating. Scale bar is 20 µm.

Fibroblasts on RGD-coupled gold squares smaller than 1 µm^2^ formed excessive numbers of lamellipodia (c.f. Fig. 4). These structures are found at the leading edge of migrating cells [7]. To investigate whether these additional lamellipodia also lead to changed motility, we plated fibroblasts on 500 nm and 2000 nm RGD-coupled gold patterns, respectively, and imaged the cells by time-lapse microscopy for the first 6 hours in culture (Fig. 7). On both patterns, it took 1–2 hours until the cells started to flatten and to form cell protrusions that were visible by phase contrast. These lamellipodia, which were present all around the cell body, were highly dynamic and visibly changed their shape and position every 2½ minutes (the time interval between individual images). Fibroblasts on the 2000 nm pattern assumed a more elongated shape 3 hours after plating. These cells also started to pull themselves along the substrate 3–4 hours after plating. In obvious contrast, cells on the 500 nm pattern flattened but retained their original non-polarized phenotype with motile circumferential protrusions. Interestingly, despite of their high number of lamellipodia, cells hardly changed their net position on this substrate. To analyze this phenomenon more precisely, we tracked cells between 3 and 6 hours after plating and determined their net translocation. For each of the two patterns 3 independent experiments were performed, and the net translocation of the nuclei of 12–15 cells was tracked per experiment. The mean speed of all cells analyzed on a respective pattern is represented in Fig. 7A. Cell tracks are depicted from one of the experiments on each pattern by tracking plots (Fig. 7B). The data show that in average, cells on the 2000 nm pattern exhibit a rate of net locomotion three times higher than on the 500 nm pattern. Light micrographs of individual cells on the 500 nm and the 2000 nm pattern, respectively, are shown at 2, 3, 4, 5 and 6 hours after plating (Fig. 7C). Note that throughout the experiment, the cell on the 500 nm pattern remained non-polarized with branching lamellipodia all around the cell body. In contrast, the cell on the 2000 nm pattern assumed an elongated shape 3–4 hours after plating, in accordance with the morphometric analysis presented earlier (cf. Fig. 4).

### Effect of RhoA/ROCK activation and inhibition on nanopattern-controlled cell shape

**Figure 8:**
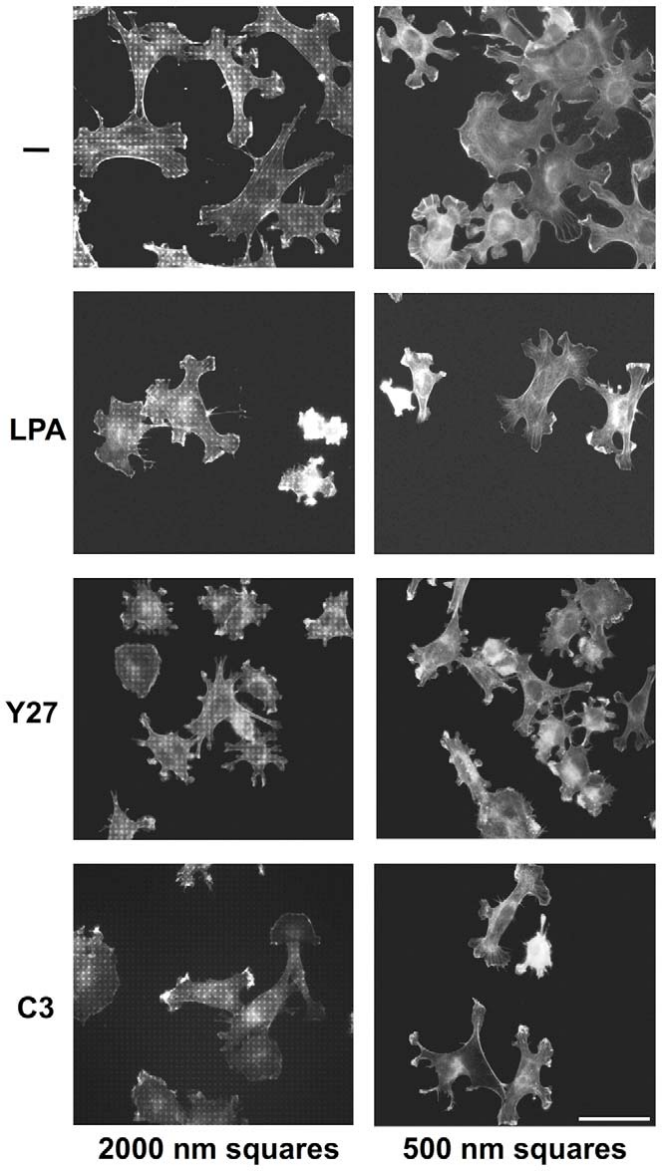
Effect of RhoA/ROCK activation and inhibition on nanopattern-controlled cell shape. Fibroblasts were seeded onto RGD-coupled 2000 nm and 500 nm gold patterns in medium with 3% fibronectin-free FCS. Cells were allowed to attach and spread for 4 hours, before medium was changed against the same medium either without drug (control) or with 5 µM lysophospatitic acid (LPA), 5 µM Y27632 (Y27), or 0.25 µg/ml C3 exoenzyme (C3), respectively. After an additional 2 hours, cells were fixed and stained for F-actin with Alexa-labeled phalloidin. Note the difference in cell morphology between the 2000 nm and the 500 nm pattern in control medium. LPA (RhoA activator), Y27632 (ROCK inhibitor) and C3 exoenzyme (Rho inhibitor) cause similar cell shape changes on both the 2000 nm and the 500 nm pattern, and morphological differences between patterns are lost. The experiment was repeated three times with comparable results; representative images from a single experiment are shown. Scale bar is 50 µm.

The inhibition of focal contact maturation and increased lamellipodia formation on 500 nm versus 2000 nm RGD-gold dots indicated that the nanopattern size might influence cell shape by controlling activation of the small GTPase RhoA and hence actin dynamics in the adhering cells (see Discussion). We therefore tested the effects of activators and inhibitors of RhoA and its downstram target Rho-dependent kinase (ROCK) on the shape and actin cytoskeleton of cells attached to the various patterns. Fibroblasts were plated on 500 and 2000 nm RGD-gold patterns for 4 hours, changed to new media without or with drugs for an additional two hours, and then fixed and stained for actin (Fig. 8). After a total of 6 hours with medium change alone, fibroblasts on the 2000 nm pattern were again largely polar with extended processes, whereas those on the 500 nm patterns showed many circumferential lamellipodia (Fig. 8, upper left panel; cf. Figs. 3 and 4). After incubation in 5 µM of the Rho activator lysophosphatidic acid (LPA), cells on both 500 and 2000 nm RGD-gold dots appeared contracted with intense actin bundles along their margins; no more obvious difference in shape could be observed between cells on the two patterns (Fig. 8, LPA). Both the specific ROCK inhibitor Y27632 (5 µM; Fig. 8, Y27) and the Rho inhibitor C3 transferase (0.25 µg/ml; Fig. 8, C3) induced collapse of long cellular processes on the 2000 nm patterns and of lamellipodia on the 500 nm pattern, with F-actin accumulating in ruffle-like structures at the cell periphery on both patterns. Again, after either Y27632 or C3 transferase treatment no apparent difference in cell shape could be observed any more between fibroblasts on the two sizes of patterns. In summary, drug induced activation as well as inhibition of RhoA/ROCK signaling appeared to eliminate the differences in cellular shape and actin cytoskeleton that are observed on the large vs. small patterns in the absence of drugs.

## Discussion

For the patterning of cell culture substrates in this work, nanostencil lithography was used rather than microcontact printing for several reasons. We had initially attempted to use microcontact printing, but found that nanostencil lithography involved fewer steps and resulted in less variation between users and experiments in the resulting patterns. Another motivating factor is that the 250 nm squares in this work are at the limit of resolution of conventional microcontact printing [21], but well within the reach of nanostencil lithography, which can produce structures ≤100 nm in size [23]. In the present study, we used patterns of RGD-coupled gold squares of different side lengths and spacings (250, 500, 1000 and 2000 nm) to restrict the growth of cell-matrix adhesions formed by adhering fibroblasts. The patterns were designed in a way that the coverage of RGD-coupled gold was always 25% of the total substrate surface, making them different only by the size and distance of adhesive spots. Thereby we could show that limiting individual cell-matrix adhesion area to <1 µm^2^ affects cell morphology, the actin cytoskeleton, fibronectin fibrillogenesis, and cell motility. The switch between two different types of cell morphologies occurred between the 500 nm and the 1000 nm pattern. Localization, size and α5-integrin content of cell-matrix adhesions suggested that fibroblasts on RGD-gold dots <1 µm^2^ only formed focal complexes, while on larger dots cells were able to convert these early adhesions into focal contacts. Adhesion maturation was accompanied by the formation of actin stress fibers, elongation of the cells, and appearance of α5-integrin-containing fibrillar adhesions as well as long fibronectin fibrils. Analysis of the morphology and migration behavior of cells on adhesive dots <1 µm^2^ showed that fibroblasts rapidly formed and retracted many lamellipodia, but failed to move in a preferred direction. This may be due to the lack of cell polarization and the absence of prominent actin stress fibers that allow the cells to retract processes at one end.

The process of cell-matrix adhesion formation and maturation has been discussed extensively [10], [28], [30]. However, little is known about specific signals emanating from the distinct stages of cell-matrix adhesions during their maturation. Several studies describe induction of lamellipodia and focal complexes by activation of Rac1 [7], [8], [31]. In our experiments, fibroblasts produced and maintained small vinculin-positive adhesions and many large lamellipodia after plating them on 250 nm and 500 nm RGD-gold patterns, indicating that the formation of focal complexes was stimulated but that their progression to focal contacts and fibrillar adhesions was inhibited. Rac1 was demonstrated to induce new cell-matrix contacts at the cell front, which are associated with the formation of lamellipodia, whereas its functional opponent RhoA serves in the maturation of focal complexes into focal contacts and in actin stress fiber formation [32]. There is increasing evidence that α5β1-integrin, coupled to its secreted ligand fibronectin, initiates RhoA activation during cell spreading [5], [25] and in response to cyclic strain of the substrate [6]. Integrin α5β1 is hardly found in focal complexes but is recruited upon their maturation into focal contacts [9]. RhoA activation through ligated α5β1-integrin induces actomyosin contraction [6], which in turn stimulates fibrillar adhesion formation and fibronectin fibrillogenesis [13]. Fibroblasts on 250 nm and 500 nm RGD-gold patterns did not show classical (>5 µm long) fibrillar adhesions. In addition, assembly of fibronectin fibrils on these patterns was clearly affected as only fine short fibrils were found. A similar finely weaved meshwork of fibronectin fibrils was observed in endothelial cells upon siRNA silencing of RhoA [33]. Based on the cited evidence, our present results indicate that on sub-micrometer RGD-gold patterns, RhoA-dependent processes are largely suppressed within the first hours of plating fibroblasts. Low levels of α5β1-integrin in cell-matrix adhesions are likely to result in low activation of RhoA, which would maintain Rac1-dependent lamellipodia formation [32]. Interestingly, we found that both maximal activation (with LPA) as well as complete inhibition (with C3 exoenzyme or Y27632) of RhoA/ROCK activity tended to eliminate the distinct morphologies of cells on the large versus the small nanopatterns that we observed in the absence of drugs. In both cases, it appeared that cells were no longer able to respond to the size differences of the nanopatterns. This finding is in accordance with a role of RhoA/ROCK-controlled actin dynamics in the process by which cells distinguish between substrate features in our model.

As mentioned, the 1000 nm and 2000 nm patterns permitted the formation of long fibrillar adhesions once focal contacts had formed on the RGD-gold dots. The likely reason for this is the fibronectin that is secreted by the adhering fibroblasts and assembled into fibrils, allowing old cell-matrix adhesion sites to be bridged and new ones to be formed. Presumably, fibronectin deficient cells [6] would not display these behaviors, but interestingly our attempts failed to get such cells to attach and spread on RGD-gold patterns (not shown). It seems possible that a total absence of fibronectin and the consequential lack of α5β1 integrin recruitment would cause a drop in activation of RhoA below the level needed for cell spreading on RGD substrates.

Micro- and nanopatterned substrates have been used extensively to explore how physical parameters of the ECM control cell behavior. For example, Chen et al. [18] produced patterns of evenly spaced fibronectin dots (3 µm diameter) by micro-contact printing to demonstrate that cell growth and survival depended on the total substrate area available for focal contact formation and spreading, rather than on the percentage of the area actually covered by ECM ligand. Xia et al. [22] extended these studies to show that asymmetric dot and stripe patterns of fibronectin can align adhering cells and direct their movements. On the lower end of the scale, Spatz and colleagues used a lithography technique to generate patterns of RGD-coupled 8 nm gold dots with variable spacings in the low nm range [16]. Their aim was to determine the minimal ligand density required for cell spreading. They showed that integrins cluster and focal contacts are able to form only if individual integrin binding sites are spaced less than 70 nm apart. With the same technique, they generated squares of RGD-gold dots similar in dimensions and spacing to our patterns, and investigated focal contact formation by REF52 fibroblasts [26]. In obvious contrast to our results, however, on 500 nm RGD-gold dot squares spaced 500 nm apart these cells formed extended paxillin-positive focal contacts that bridged the gaps between patches. Thus, patterns in the sub-micrometer range did not restrict the growth of cell-matrix adhesions in their case. This is probably due to the different cell types used: REF52 cells are considerably larger and spread more extensively than immortalized mouse embryo fibroblasts which resemble primary cells [34].

In another study directly relevant to our present, Lehnert et al. [21] used micro-contact printing to produce patterns of fibronectin squares with side lengths from 0.3–3 µm (∼0.1–10 µm^2^) and center-to-center spacing from 1–30 µm. They found that a pattern of 0.25 µm^2^ fibronectin dots with 5 µm spacing (1% coverage of the substrate with ligand) was about the limit for B16 melanoma cells to establish focal contacts, to spread, and to generate actin stress fibers. These authors observed cell spreading and small cell-matrix adhesions even on 0.1 µm^2^ fibronectin dots when the spacing was reduced to 1 µm; however, they did not investigate cell motility and the actin cytoskeleton under these conditions. In the present study, we tested similar patterns engineered with the nanostencil technique. We however kept the area covered with ligand constant at 25% and used RGD peptide instead of fibronectin, in order to reduce integrin-α5β1 and RhoA activation during initial attachment of cells [5]. Our present results show that for mouse embryo fibroblasts, adhesive RGD-gold patterns in the sub-micrometer range inhibit the maturation of focal complexes to focal contacts as well as stress fiber assembly, but still promote extensive lamellipodia formation and cell spreading. This indicates a difference in the balance between Rac1 and RhoA activation in cells on the various patterns, which at present is difficult to demonstrate directly due to the low number of cells available in our experiments. Nevertheless, our findings demonstrate the potential of the nanostencil patterning technique for manipulating cell-matrix adhesion sites and hence the shape and motility of cells adhering to engineered surfaces. RhoA-regulated cell shape has been reported to control the differentiation of mesenchymal stem cells towards the adipocyte and osteoblast lineage, respectively [20]. Thus in the future, the nanostencil method may offer new possibilities to control more precisely the interaction of mesenchymal cells with implant surfaces, and to influence their differentiation around the implant.

## Materials and Methods

### Nanostencil technique

Nanostencil lithography is essentially a shadow-masking technique in which a material is added or removed through nanoscale apertures in a thin membrane to create corresponding nanopatterns on a substrate in conformal contact. In this case, Au nanopatterns were evaporated onto glass coverslips.

Precise details on nanostencil fabrication can be found in [35] but we describe the major steps here. Fabrication begins by depositing a 200 nm thick silicon nitride film on both faces of a polished Si wafer. The nanoapertures are patterned into a resist on the first face by electron beam lithography, and transferred into the nitride film by reactive ion etching. Large (0.5×0.5 or 1×1 mm) ‘windows’ are patterned onto the second face using photolithography and reactive ion etching. The exposed Si at the ‘window’ in the second face is etched through the wafer using a combination of reactive ion and wet chemical etching, until it meets the nanopatterned nitride film. The result is a 200 nm thick nanopatterned nitride membrane supported in a sturdy silicon frame. The wafer is cleaved into chips to facilitate stencil handling.

Patterning occurred in the following steps. The glass coverslips were affixed to a large glass wafer using polyimide tape. Next, the stencils were affixed to the coverslips using polyimide tape. The assembly of wafers, coverslips, and nanostencils was cleaned in an oxygen plasma chamber (30 s, 500 W) to ensure proper metal film adhesion to the coverslips (Tepla 300, PVA Tepla AG, Germany). Finally, the assembly was placed in a LAB 600 electron beam evaporator (Leybold Optics GmbH, Germany) for metallization. First, a 5 nm thick Ti layer was deposited to ensure the Au would stick to the coverslip. Next, 40 nm of Au was deposited to serve as the cell adhesive pattern. Following the evaporation the nanostencils were removed from the coverslips, and the coverslips were removed from the glass wafer and stored for use.

### RGD-coupling of gold patterned coverslips

Patterned coverslips were cleaned in a UV-ozone photoreactor (PR-100, UVP, Upland, CA) for 30 minutes. Each coverslip was then placed in a μ-dish (35 mm diameter, high) from Ibidi (Vitaris, Baar, Switzerland) and subsequently coated with a solution of the RGD peptide Ac-Gly-Cys-Gly-Arg-Gly-Asp-Ser-Pro-Gly-NH2 (Polypeptide, Boulogne, France) at 3 mg/ml in water for 24 hours. Patterns were then washed 3 times with PBS and passivated with PLL(20)-g[3.5]-PEG(2) (SuSoS AG, Dübendorf, Switzerland) in water at 0.2 mg/ml for 2 hours. PLL-g-PEG solution was extensively washed away with PBS and the PBS was then again changed against Dulbecco's modified Eagle medium (D-MEM; Seromed, Basel, Switzerland).

### Plating of cells

A clonal mouse embryonic kidney fibroblast cell line immortalized by stable transfection with SV40 large T antigen [36] was obtained from Dr. Reinhard Fässler (Max Planck Institute for Biochemistry, Martinsried, Germany). Cells were maintained at 37°C with 6% CO_2_ in Dulbecco's modified Eagle medium (D-MEM; Seromed, Basel, Switzerland) containing 10% fetal calf serum (FCS; Gibco/Invitrogen, Basel, Switzerland). Instead of a commercial established fibroblast line e.g. NIH-3T3, we chose these cells for our experiments because they are not transformed and resemble primary fibroblasts in their gene expression pattern [34] and integrin profile [6]. Cells were harvested with trypsin-EDTA (Gibco/Invitrogen, Basel, Switzerland), resuspended in D-MEM containing 3% fibronectin-depleted FCS [6], and seeded onto the nanopatterned substrates (40'000 cells per 35 mm dish). The medium was depleted of fibronectin to reduce deposition of this adhesive glycoprotein to the passivated areas. Dishes were kept at 37°C and 6% CO_2_ for 4 hours. In certain experiments, medium was changed to D-MEM/3% fibronectin-free FCS containing 5 µM lysophophatidic acid (LPA, Sigma), 5 µM Y27632 (Y27; Calbiochem), or 0.25 µg/ml bacterial recombinant C3 transferase (C3; Cytoskeleton), respectively, and cells were incubated for an additional 2 hours. Optimal drug concentrations for these cells had been determined in previously published experiments [3], [6]. Cells were then fixed with 4% paraformaldehyde for 10 minutes, followed by washing with PBS.

### Control experiments for RGD functionalization

In control experiments (Figure S1), gold-patterned coverslips were coated with 3 mg/ml of a peptide in which the RGD sequence was changed to RDG (Ac-Gly-Cys-Gly-Tyr-Gly-Arg-Asp-Gly-Ser-Pro-Gly-NH2; synthesized by J. Patterson in the lab of J. A. Hubbell, Ecole Polytechnique Féderale de Lausanne). Additionally, patterns were functionalized as above with active RGD and control RDG peptide mixed in different ratios (1∶1, 1∶4 and 1∶9), keeping the total concentration of 3 mg/ml constant. After passivation with PLL-g-PEG, cells were plated as above before fixation. We observed that fibroblasts spread fully on gold patterns coupled with pure RGD as well as with mixtures of RGD∶control peptide of 1∶1 and 1∶4. On patterns coated with 1∶9 mixtures, cells spread partially but did not polarize, whereas on plain control peptide they attached but did not spread (Fig. S1). These results showed that with pure RGD peptide and up to a 1∶4 dilution with control peptide, gold patterns were functionally saturated with RGD ligand for cell spreading.

### Immunofluorescence and phalloidin staining

Patterned cover slips with attached, fixed cells were permeabilized at room temperature for 30 minutes in PBS solution containing 3% BSA and 0.1% Triton X-100 (Sigma, Buchs, Switzerland). Cells were then stained for 1 hour at room temperature with one or several of the following reagents in PBS containing 3% BSA and 0.1% Triton X-100: anti-fibronectin antiserum [37] diluted 1∶300; anti-α5-integrin diluted 1∶500 (BD Pharmingen, Basel, Switzerland); anti-vinculin diluted 1∶1000 (Sigma, Buchs, Switzerland); Alexa 546-labeled phalloidin at 1 µg/ml (Sigma, Buchs, Switzerland). After staining, cells were washed three times with PBS/0.1% Triton X-100 and incubated for 1 hour with one or several of the following secondary antibodies: Alexa 488-labeled goat anti rabbit IgG; Alexa 488-labeled goat anti mouse IgG; Alexa 488-labeled goat anti rat IgG; Alexa 633-labeled goat anti rabbit (all from Sigma, Buchs, Switzerland) diluted 1∶1000 in PBS/3% BSA/0.1% Triton X-100. Cells were again washed three times with PBS/0.1% Triton-X100 and mounted in Prolong Gold antifade reagent (Invitrogen, Basel, Switzerland). Slides were examined with a Zeiss Z1 or a Olympus BX51 fluorescence microscope equipped with 20×/0.8 and 40×/0.75 objectives and filter cubes for Alexa 488 and Alexa 546. Numbers of lamellipodia, cell span, number and length of actin and fibronectin fibers per cell were determined from fluorescence images of cells on the various patterns. At least 30 cells from three independent experiments were measured per pattern. Data were evaluated by ANOVA to test the null hypothesis, and the significance of differences between 500 nm and 1000 nm patterns was determined by unpaired Student's t-test.

### Live Imaging Microscopy

For live imaging microscopy, fibroblasts were seeded into Ibidi μ-dishes (Vitaris, Baar, Switzerland) onto RGD-coupled gold patterns as described above. Immediately after seeding, cells were observed with a Zeiss inverted microscope for the following 6 hours. Time-lapse phase contrast images were collected every 2½ minutes. Pictures were processed and analyzed with the ImageJ program and the manual tracking plug-in. For quantification of cell velocity, ≥40 cells per pattern were measured, and the significance of the difference between 2000 nm and 500 nm patterns was determined by unpaired Student's t-test.

## Supporting Information

Figure S1 **Control experiment for RGD functionalization of gold patterns.** 1000 nm gold patterns were coupled with either RGD peptide alone (pure RGD), control peptide alone (pure RDG control), or mixtures of RGD∶control peptide of 1∶1, 1∶4 and 1∶9, respectively (total peptide concentration 3 mg/ml in all cases). Fibroblasts were plated, fixed and stained for F-actin as described in Materials and Methods. Note that cells spread fully on the coated gold patterns up to a RGD∶control peptide ratio of 1∶4. The experiment was repeated three times with comparable results; representative images from a single experiment are shown. Scale bar is 50 µm.
(TIF)
