# Supplementary figures and images for: Nano-Stenciled RGD-Gold Patterns That Inhibit Focal Contact Maturation Induce Lamellipodia Formation in Fibroblasts
Source: PLoS One. 2011 Sep 27;6(9):e25459. doi: 10.1371/journal.pone.0025459 (PMC3181263; doi:10.1371/journal.pone.0025459)

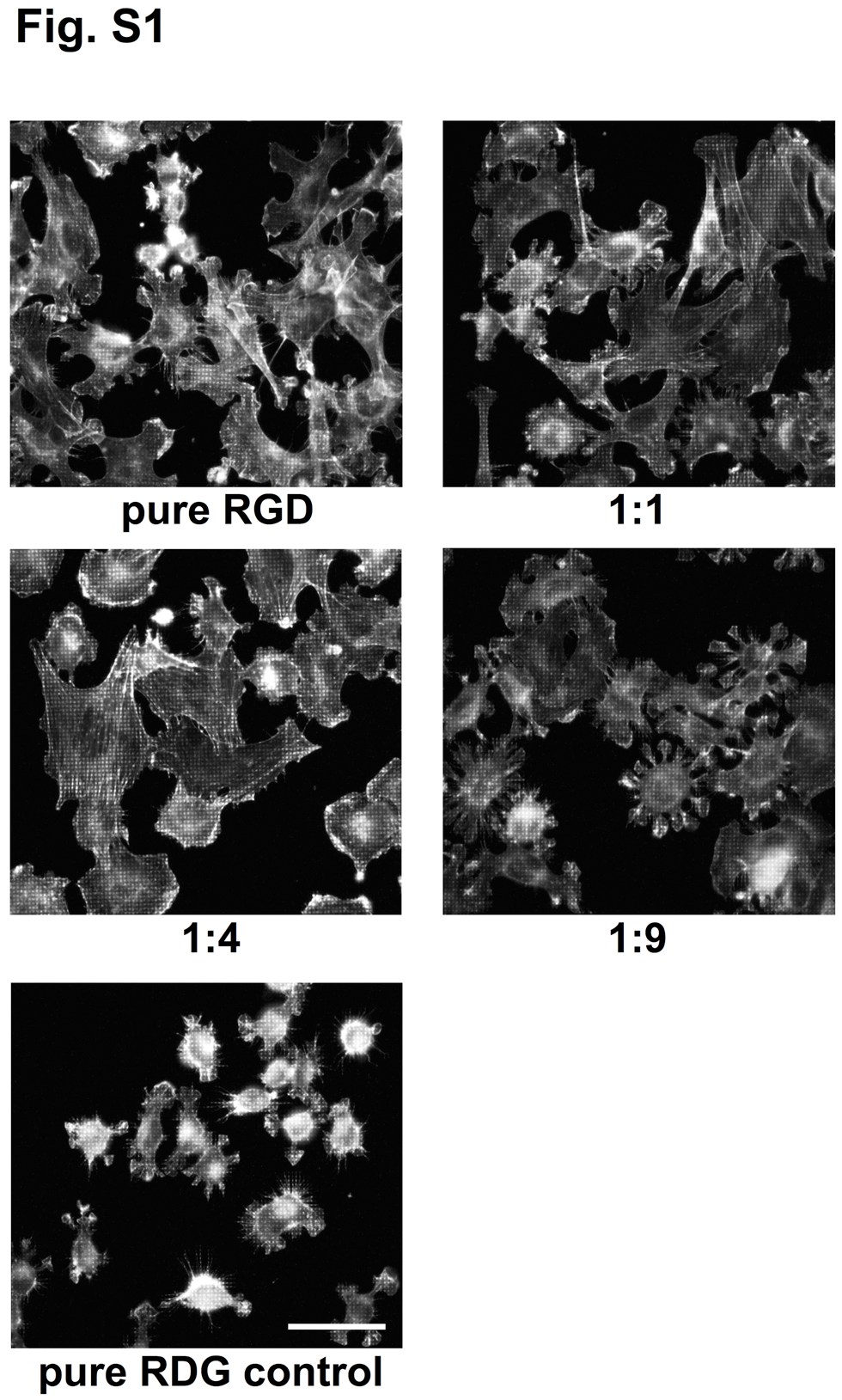

Supplement: Figure S1 — Control experiment for RGD functionalization of gold patterns. 1000 nm gold patterns were coupled with either RGD peptide alone (pure RGD), control peptide alone (pure RDG control), or mixtures of RGD∶control peptide of 1∶1, 1∶4 and 1∶9, respectively (total peptide concentration 3 mg/ml in all cases). Fibroblasts were plated, fixed and stained for F-actin as described in Materials and Methods. Note that cells spread fully on the coated gold patterns up to a RGD∶control peptide ratio of 1∶4. The experiment was repeated three times with comparable results; representative images from a single experiment are shown. Scale bar is 50 µm. (TIF) [file pone.0025459.s001.tif]
